# Effects of Aluminum Oxide Nanoparticles on the Growth, Development, and microRNA Expression of Tobacco (*Nicotiana tabacum*)

**DOI:** 10.1371/journal.pone.0034783

**Published:** 2012-05-11

**Authors:** Caitlin E. Burklew, Jordan Ashlock, William B. Winfrey, Baohong Zhang

**Affiliations:** Department of Biology, East Carolina University, Greenville, North Carolina, United States of America; National Taiwan University, Taiwan

## Abstract

Nanoparticles are a class of newly emerging environmental pollutions. To date, few experiments have been conducted to investigate the effect nanoparticles may have on plant growth and development. It is important to study the effects nanoparticles have on plants because they are stationary organisms that cannot move away from environmental stresses like animals can, therefore they must overcome these stresses by molecular routes such as altering gene expression. microRNAs (miRNA) are a newly discovered, endogenous class of post-transcriptional gene regulators that function to alter gene expression by either targeting mRNAs for degradation or inhibiting mRNAs translating into proteins. miRNAs have been shown to mediate abiotic stress responses such as drought and salinity in plants by altering gene expression, however no study has been performed on the effect of nanoparticles on the miRNA expression profile; therefore our aim in this study was to classify if certain miRNAs play a role in plant response to Al_2_O_3_ nanoparticle stress. In this study, we exposed tobacco (*Nicotiana tabacum*) plants (an important cash crop as well as a model organism) to 0%, 0.1%, 0.5%, and 1% Al_2_O_3_ nanoparticles and found that as exposure to the nanoparticles increased, the average root length, the average biomass, and the leaf count of the seedlings significantly decreased. We also found that miR395, miR397, miR398, and miR399 showed an extreme increase in expression during exposure to 1% Al_2_O_3_ nanoparticles as compared to the other treatments and the control, therefore these miRNAs may play a key role in mediating plant stress responses to nanoparticle stress in the environment. The results of this study show that Al_2_O_3_ nanoparticles have a negative effect on the growth and development of tobacco seedlings and that miRNAs may play a role in the ability of plants to withstand stress to Al_2_O_3_ nanoparticles in the environment.

## Introduction

Nanoparticles are classified as being materials in which at least one dimension of the material is less than 100 nanometers in diameter [1]. Nanoparticles are becoming an area of research interest due to their unique properties, such as having increased electrical conductivity, ductility, toughness, and formability of ceramics, increasing the hardness and strength of metals and alloys, and by increasing the luminescent efficiency of semiconductors [2]. Nanoparticles are heavily used in an industrial setting because they can be used to manufacture lightweight, strong materials as well as acting as pigments in products such as paints, sunscreens, and cosmetics [1]. Because nanoparticles have a large surface area to volume ratio, the use of nanoparticles in both industry and daily life is greatly increasing in realms that include advancing the quality of everyday materials and processes, improving the function of electronics and information technology, allowing more sustainable energy applications, and acting as key players in environmental remediation applications [1]. Aluminum oxide is also very wear-resistant, has good thermal conductivity, resists strong acid and alkalai containing materials, is easily shaped, and has high strength and stiffness which makes it a prime material to use in making products that include high temperature electrical insulators, high voltage insulators, thermometry sensors, wear pads, ballistic armor, and grinding media (http://accuratus.com/alumox.html). Because the amount of nanoparticles that are used in industry is drastically increasing as more useful ways of integrating them into products is becoming evident, the amount of nanoparticles that are released into the environment and the effects these nanoparticles may have needs to be assessed. Nanoparticles have been highly used in the past 20 years, but it has only been recently that any organizations have dedicated money and research to quantifying the amount of nanoparticle residues that may be found in the environment and the potential risks they may have. Recently, the amount of research funding dedicated to nanoparticle environmental health and safety increased from $35 million in 2005, to $117 million in 2011 [1]. Although more research funding is being provided for nanoparticle environmental health and safety research, to date, few experiments have been performed to show the effects nanoparticles may have on the growth, development, and gene expression in plants. It is unknown that the regulatory mechanism under nanoparticle exposure. microRNAs (miRNAs), are a newly discovered highly-conserved, endogenous class of regulatory molecules that do not code for proteins [3], [4]. miRNAs are approximately 20–22 nucleotides in length and work in post transcriptional gene regulation by either targeting messenger RNAs (mRNAs) for degradation, or by inhibiting the translation of mRNAs [4]. miRNAs have been shown to aid in the regulation of many processes within plants such as leaf and root development, organ maturation, cell proliferation, flowering time, and abiotic stress response [5], [6]. Recent studies have shown that miRNAs help to mediate the expression of more than 30% of protein coding genes [7], [8] and this number is expected to increase as more miRNAs are discovered and their target mRNAs are identified.

In this study, we employed tobacco as a model species to investigate the effects of aluminum oxide nanoparticles on the growth and development in agricultural plants and to investigate the potential role of miRNAs during this process. The results of this study show that as concentrations of aluminum oxide nanoparticles increase, the root length, the average biomass, and the leaf count of each tobacco seedling decreased and the expression profile of certain miRNAs was significantly up regulated.

## Materials and Methods

### Seed Sterilization, Media Preparation, and Tobacco Treatment

Tobacco (*Nicotiana tabacum*) seeds were sterilized by soaking in 70% ethanol for two minutes, followed by soaking in 10% bleach for 15 minutes. The seeds were then rinsed with sterilized water approximately four times until no bleach odor remained. After each rinsing with water, the immature tobacco seeds that floated were removed and discarded to ensure mature seeds were being sowed on Petri dishes. The basic medium used for growing tobacco contained the following: 0.44 g Murashige and Skoog (MS) salts supplemented with 1× Gamborg's B5 Vitamins, 1 g sucrose, and 0.8 g agar per 100 mL of media. Four concentrations (0, 0.1, 0.5 and 1.0%) of Al_2_O_3_ nanoparticles were also added to the media before the pH was adjusted. The pH of the media was adjusted to approximately 5.8 after the addition of all media components. After media preparation, 25 sterilized tobacco seeds were sowed on each Petri dish, for a total of 20 plates (5 plates per Al_2_O_3_ nanoparticle concentration). The plates were subsequently placed under a 16 h day/8 h night cycle at room temperature for exactly 3 weeks.

### Total RNA Extraction

Three week old tobacco seedlings were harvested from their respective plates and were frozen in liquid nitrogen after physical measurements were recorded such as root length, leaf count, germination rate, and average seedling biomass. The seedlings were placed in a −80°C freezer until total RNA extraction. Total RNA was extracted using the mirVana miRNA Isolation Kit (Ambion, Austin, TX) according to the manufacturer's protocol. The total RNA was then quantified and the quality was assessed by using a Nanodrop ND-1000 (Nanodrop Technologies, Wilmington, DE) and RNA samples were stored in a −80°C freezer until further use and analysis.

### Analyzing microRNA Expression Variations Using RT-PCR and qRT-PCR

Applied Biosystems TaqMan miRNA Assays were used to detect and to quantify the miRNAs in tobacco using a stem-loop real-time PCR according to the manufacturer's instructions. There were two steps in the TaqMan Assays which included reverse transcription of the mature miRNA into a longer single-stranded cDNA sequence using a miRNA-specific stem-looped primer and secondly, quantitative real-time PCR. In brief, a single-stranded miRNA cDNA sequence was generated from 1 µg of the total RNA collected from each treatment (0%, 0.1%, 0.5%, and 1%). The cDNA was generated using reverse transcription with the Applied Biosystems TaqMan microRNA Reverse Transcription Kit and the miRNA-specific stem-looped RT primers provided with the kit. Previous studies have shown that miR156, miR157, miR159, miR162, miR167, miR169, miR172, miR395, miR396, miR397, miR398, and miR399 are important for plant growth as well as environmental stress response, therefore these miRNAs were selected to investigate the effect nanoparticle exposure has on tobacco. We also used two stress related genes, alcohol dehydrogenase (ADH) and ascorbate peroxidase (APX), to investigate the effects nanoparticle exposure has on tobacco. In the relative quantification analysis, tubulin, a housekeeping gene, was used as a reference gene in order to normalize expression values. Three biological replicates were run for each gene within each treatment and each biological replicate was technically duplicated to minimize the chance of error. The results were analyzed using the ΔΔC_T_ method.

### Statistical Analysis of the Physical Characteristics and miRNA Fold Changes in Tobacco

After the physical characteristics of tobacco were measured and the fold changes in the miRNA expression levels in tobacco were quantified, the results were analyzed using the statistical software SPSS version 19. For each measurement (i.e. root length, leaf count, etc.), the measurements found for all four treatments were tested for their significance using a one-way ANOVA test with a LSD post-hoc test. All treatments were tested with a 95% confidence interval.

## Results

### Aluminum oxide nanoparticles affected the growth and development of tobacco seedlings

Once the tobacco seeds were plated, it took approximately three to five days for them to germinate. After ten days, the germination rates of the tobacco seedlings were calculated for each concentration of aluminum oxide nanoparticles. For the seedlings grown on control media without any aluminum oxide nanoparticles, the average germination rate was 99.2%, therefore almost all of the tobacco seeds germinated. In the media containing 0.1% aluminum oxide nanoparticles, the average germination rate was 92.8% and the seeds grown in 0.5% aluminum oxide nanoparticles had an average germination rate of 96.0%. Lastly, the seeds grown in 1.0% aluminum oxide nanoparticles had a germination rate that was much lower than the previous three concentrations at 88% (Table 1). Although there was some variation between the germination rates of seeds grown in media with differing concentrations of aluminum oxide nanoparticles, this variation was not statistically significant, therefore aluminum oxide nanoparticles did not significantly affect the germination rate of tobacco seedlings.

**Table 1:** Nanoparticles effect the growth and development of three week old tobacco seedlings.

	MS Media (Control)	0.1% Al_2_O_3_ Nanoparticles	0.5% Al_2_O_3_ Nanoparticles	1% Al_2_O_3_ Nanoparticles
**Average Root Lengths (mm)**	29.54±2.09 a	22.04±3.01 b	5.52±0.24 c	2.31±0.13 c
**Average Leaf Count**	3.77±0.09 a	3.34±0.14 b	2.82±0.08 c	2.39±0.14 d
**Average Germination Rate**	24.80±2.33 a99.2%	23.20±1.24 a92.8%	24.00±0.55 a96%	22.00±1.00 a88%
**Average Biomass per seedling (mg)**	12.0±0.0015 a	7.5±0.0015 b	2.8±0.00056 c	2.1±0.0003 c

All physical measurements were averaged for each treatment (control, 0.1% Al_2_O_3_, 0.5% Al_2_O_3_, and 1% Al_2_O_3_). Each average is stated with the standard error associated with that treatment and the significance levels are denoted with the letters, a–d. Different letters dentote statistical significance within the physical measurement.

**Figure 1:**
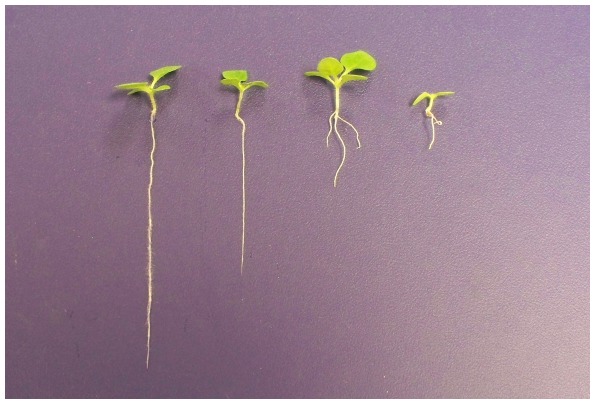
Phenotype of aluminum oxide treated tobacco seedlings. From left to right, control, 0.1%, 0.5%, and 1% aluminum oxide treated seedlings.

After three weeks of culture, the seedlings grown in the presence of aluminum oxide nanoparticles showed an obvious decrease through the visualization of the root lengths, leaf count, and biomasses of the seedlings (See Table 1 and Figure 1). In this experiment, root growth and development seemed to be affected the most with a statistically significant decrease in root length as the concentration of aluminum oxide nanoparticles increased. For the control seedlings, the average root length was 29.5 mm. For the seedlings exposed to 0.1% aluminum oxide nanoparticles, the average root length decreased to 22.0 mm, which was significantly lower than the control (p<0.05). The seedlings exposed to 0.5% and 1.0% aluminum oxide nanoparticles had average root lengths of 5.5 mm and 2.3 mm, respectively, which were both significantly lower than the control (p<0.01) and the seedlings grown in 0.1% aluminum oxide nanoparticles (p<0.01). Although the seedlings grown in 0.5% and 1.0% aluminum oxide nanoparticles were significantly lower than the control and 0.1% aluminum oxide seedlings, the difference in root length between each other was not highly significant (p<0.05). It should also be noted that as the concentration of aluminum oxide nanoparticles increased, the probability of the seedlings forming multiple roots also increased (data was not shown). Overall, as the concentration of aluminum oxide nanoparticles increased, the average root lengths of three week old tobacco seedlings significantly decreased (Figure 1).

In this experiment, aluminum oxide nanoparticles affect the amount of leaves three week old tobacco seedlings have (Table 1). For the control, the average leaf count was 3.77. For the 0.1% and 0.5% aluminum oxide seedlings the average leaf count was 3.36 and 2.82, respectively. For the 1.0% aluminum oxide seedlings, the average leaf count was 2.39. Our results show that as the concentrations of aluminum oxide nanoparticles increased, the amount of leaves per seedling also significantly decreased. As a result, aluminum oxide nanoparticles have a negative effect on the growth and development of three week old tobacco seedling leaves.

Lastly, the average biomass per tobacco seedling was assessed to see if aluminum oxide nanoparticles had any significant affect on the growth of tobacco seedlings. For the seedlings grown on control media, the average biomass per seedling was calculated to be 11.5 milligrams. For the 0.1%, 0.5%, and 1% aluminum oxide exposed seedlings, the average biomass per seedling was calculated to be 7.5 milligrams, 2.8 milligrams, and 2.1 milligrams, respectively (Table 1). The average biomass of the control, 0.1%, and 0.5% aluminum oxide seedlings all significantly decreased (p<0.05) as the concentration of the nanoparticles increased. The average biomass of the seedlings grown in 1% aluminum oxide were significantly lower (p<0.05) than the control and 0.1% aluminum oxide seedlings, but was not significantly lower than the seedlings grown in 0.5% aluminum oxide nanoparticles. Overall, the average biomass of three week old tobacco seedlings decreased as the exposure to aluminum oxide nanoparticles increased.

### Aluminum oxide nanoparticles alter microRNA expression in tobacco

**Figure 2:**
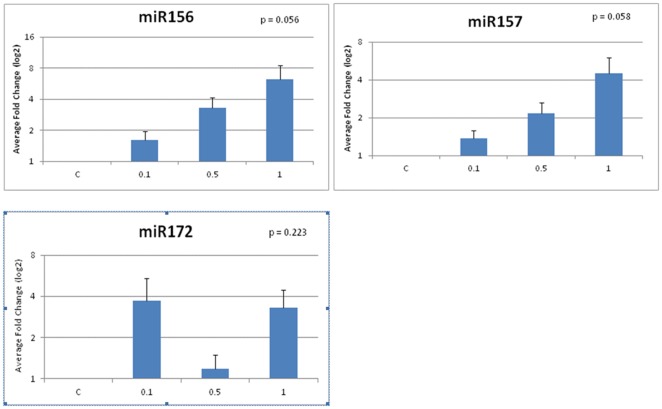
Average miRNA fold changes in expression in response to aluminum oxide nanoparticles (control, 0.1%, 0.5%, and 1%) that do not fall within the 95% confidence interval.

**Figure 3:**
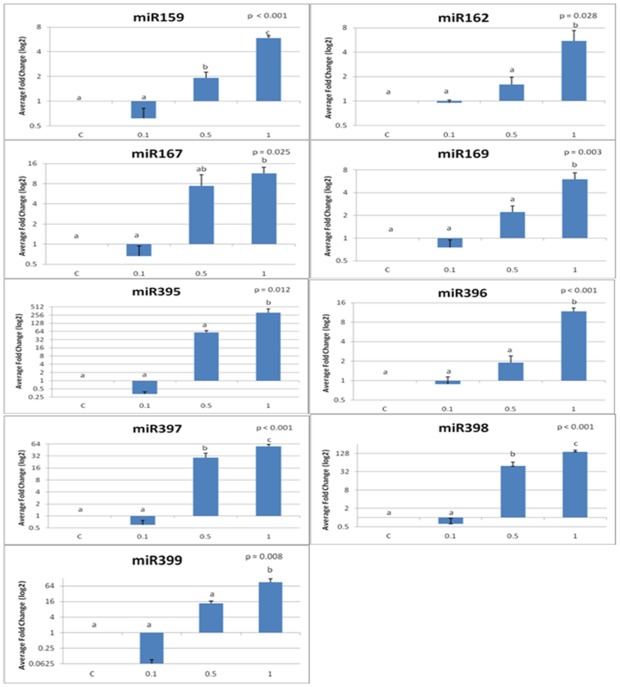
Statistically significant average fold changes of microRNA expression levels in tobacco plants exposed to differing treatments of aluminum oxide nanoparticles. (control, 0.1%, 0.5%, and 1%). Statistical significance is denoted by letters a–d. Different letters signify statistical significance.

Aluminum oxide nanoparticles significantly altered the expression levels of certain miRNAs in tobacco. In the tobacco plants exposed to 0.1% aluminum oxide nanoparticles, all of the tested miRNAs were down regulated except for miR156, miR157, and miR172 which were up regulated (Figure 2). However, in the plants exposed to 0.5% and 1% aluminum oxide nanoparticles, all of the miRNAs that were tested were up regulated. The expression of three miRNAs (miR156, miR157, and miR172) was up regulated in response to increasing aluminum nanoparticle exposure, but these fold changes in expression were not significantly significant (p>0.05) (Figure 2). These three miRNAs had average fold changes less than 10. Nine miRNAs (miR159, miR162, miR167, miR169, miR395, miR396, miR397, miR398, and miR399) had statistically significant increases in fold change of expression (Figure 3). miR159, miR162, miR167, and miR169 were significantly up regulated with fold changes in the 1% Al_2_O_3_ nanoparticle treatment of 5.9, 5.5, 11.4, and 6.0 fold, respectively. Of all significantly up regulated miRNAs, four miRNAs showed greater fold changes in the 1% Al_2_O_3_ concentration than the others. These included miR395, miR397, miR398, and miR399 with average fold changes in expression of 315, 55, 144, and 90, respectively in response to 1% Al_2_O_3_ nanoparticles.

### Aluminum oxide nanoparticles alter the expression of stress-related genes in tobacco

**Figure 4:**
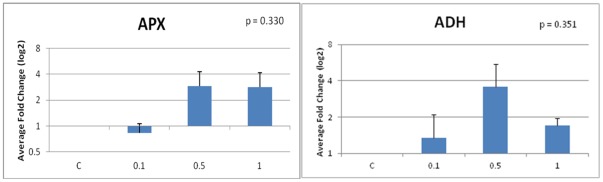
Average fold changes in expression of two stress genes, APX and ADH in tobacco plants exposed to aluminum oxide nanoparticles (control, 0.1%, 0.5%, and 1%).

In this study, we also observed a change in the expression levels of two stress related genes, ascorbate peroxidase (APX) and alcohol dehydrogenase (ADH). The trends for the expression levels in APX and ADH were slightly different from the trends in expression for the miRNAs. For APX, the expression was down regulated 0.83 fold in the 0.1% treatment, up regulated 2.89 fold in the 0.5% treatment, and then slightly decreases to 2.82 fold in the 1% treatment (Figure 4). For ADH, the fold change is up regulated to 1.34 fold and 3.57 fold in the 0.1% and 0.5% concentration, but decreases to 1.70 fold in the 1% treatment in a similar fashion to APX. For both APX and ADH, the fold change was not statistically significant (Figure 4).

## Discussion

Nanoparticles have been increasingly used in the past 20 years due to their unique properties such as in electronics, biomedical applications, pharmaceuticals, cosmetics, energy applications, and materials [9]; therefore the potential for nanoparticles to contaminate the environment is much greater than in the past. Because plants are sessile unlike animals, they must cope with environmental stressors in different ways than simply the act of relocation. Plants typically respond to environmental biotic and abiotic stress through molecular routes. In the past decade, research has been conducted on miRNAs and they are believed to be an ancient form of post transcriptional gene regulation because of their high conservation [10]. Because miRNAs mediate gene expression, they are thought to be one of the key factors in plant stress response. In this study, we analyzed the effects of aluminum oxide nanoparticle exposure on the miRNA expression levels in tobacco seedlings as well as how nanoparticles affect the growth and development of tobacco. We found that as aluminum oxide nanoparticle concentrations increased, the root lengths, leaf count, andthe overall biomass of each seedling significanly decreased. We also found that as the concentration of aluminum oxide nanoparticles increased, the general miRNA expression profile of selected miRNAs was up regulated.

The results of our study showed that the germination rate of tobacco seedlings exposed to increasing amounts of aluminum oxide nanoparticles was not statistically significant. Our results were consistent with a study which investigated the effects of five different types of nanomaterials (multi-walled carbon nanotubules, aluminum oxide, zinc oxide, aluminum, and zinc) on the seed germination rate and root length of five agriculturally important crops (radish, rape, ryegrass, lettuce, corn, and cucumber) [11]. They found that out of all five crops, that only the seed germination rate of ryegrass and corn were affected by nano-Zn and nano-ZnO, respectively. In short, Lin and Xing (2007) found that in response to 200 mg/L Al_2_O_3_ nanoparticles that none of the germination rates from all 5 plants they tested were significantly affected. These results mirror our results because 2000 mg/L converts to the concentration of 0.2 g of Al_2_O_3_ nanoparticles per 100 mL of media. This concentration fits approximately in the middle of the range of Al_2_O_3_ nanoparticle concentrations that we used (0.1 g, 0.5 g, and 1 g of Al_2_O_3_ nanoparticles per 100 mL of media). Lin and Xing (2007) state that the seed coat of the tobacco seeds were most likely not permeable to the aluminum oxide nanoparticles, therefore the germination rate was not affected greatly. Thus, it would not be until after the seedlings started to emerge from the seed coat, that they would be affected by aluminum oxide nanoparticles.

Numerous studies over the past few decades have shown that the primary mode of toxicity related to exposure to aluminum ions (Al_3_) is preventing root elongation in plants [12], [13], [14]. Aluminum compromises approximately 7% of the earth's crust though most forms of aluminum are bound by ligands that make them less toxic [12]. Although most forms of aluminum in soil are not very toxic, aluminum solubility increases as the pH of water decreases, therefore more soluble aluminum is found in acidified soils. Since approximately 40% of arable land is considered acidified, aluminum toxicity research is in great demand as it plays a large role in the potential growth of crops [15]. Although much is known about the toxicity of aluminum ions, not much is known about the toxicity that aluminum oxide nanoparticles may have on plants, therefore one aim of this project is to assess whether aluminum oxide nanoparticles also affect the growth and development of plants.

In this study, we also found that as aluminum oxide nanoparticles increased, the average lengths of three week old tobacco seedling roots decreased. Between the control seedlings and the 0.1% aluminum oxide nanoparticle-treated seedlings, the average root length decreased by 25.4%. The 0.5% and 1.0% aluminum oxide-treated seedlings decreased in length by 81.4% and 92.2%, respectively, as compared to the control. As a result, we were able to give evidence that aluminum oxide nanoparticles have a negative effect on the growth of the roots of three week old tobacco seedlings. The phytotoxicity of other metal nanoparticles has also been evaluated through other studies. Aluminum oxide nanoparticles have been shown in previous studies to inhibit root elongation in corn, cucumber, soybean, cabbage, and carrot [11]. Other studies have shown that metal nanoparticles negatively affect the root elongation in plants as well [11], [16], [17]. Zinc oxide nanoparticles have been shown to greatly decrease the root lengths of ryegrass, radish, rape, lettuce, and cucumber [11], [18]. The study of Lin and Xing (2008) also shows that the most likely contributing cause to the decrease in root growth was from damage to epidermal and cortical cells in the roots of ryegrass by zinc oxide nanoparticles. A study by Asli and Neumann (2009) [17] showed that titanium dioxide nanoparticles interfered with the ability of maize to uptake water in the roots by forming aggregates along the root cell walls. This ultimately blocks water uptake and therefore led to reduced root development. As a result, aluminum oxide nanoparticles may have aggregated along the roots of the tobacco seedlings, therefore impeding their proper water uptake and ultimately affecting their growth and development, but more research would need to be conducted in order to confirm the exact mode of damage.

We also found that as the concentration of aluminum oxide nanoparticles increased, the average biomass of each three week old seedling decreased (See Table 1). The biomass for the seedlings exposed to 0.1%, 0.5%, and 1% aluminum oxide nanoparticles significantly decreased as nanoparticle concentration increased. This drastic change in the reduction of seedling biomass is most likely correlated to the decreasing lengths of roots. Because of the reduction in root length and leaf count as nanoparticle concentration increases, the biomass would naturally decrease as well because seedlings at this stage only consist of the primary root and leaves. Little is known about the uptake and translocation of nanoparticles and their subsequent effect on the growth and development of plants, therefore additional studies are needed to determine the mechanisms that aluminum oxide nanoparticles have on the phytotoxicity of tobacco seedlings.

miRNAs are a newly discovered class of small regulatory RNAs that act in post transcriptional gene regulation by either marking mRNAs for cleavage or by preventing ribosomal translation of the messenger RNA into a protein [3], [4]. miRNAs are highly conserved, therefore they are thought to play a major role in biotic and abiotic stress responses. miRNAs have been shown to play key roles in plant response to many environmental stressors such as drought [19], [20], salinity [21], [22], and heavy metals [23], [24], [25]. In this experiment, our goal was to identify a few miRNAs that may be involved in plant stress response to aluminum oxide nanoparticles. We chose miR156, miR157, miR159, miR162, miR167, miR169, miR172, miR395, miR396, miR397, miR398, and miR399 because they are well known miRNAs that already have known functions in plants, but our aim was to see if they may have alternative uses in abiotic stress response to aluminum oxide nanoparticles. Our results show that nine miRNAs were significantly up regulated (miR159, miR162, miR167, miR169, miR395, miR396, miR397, miR398, and miR399) as the concentration of aluminum oxide nanoparticles increased. It also needs to be noted that in all of these miRNA expression profiles, the nine previously mentioned miRNAs are down regulated in expression at the 0.1% aluminum oxide nanoparticle concentration. It is not known why this trend occurred throughout all nine expression profiles, but miRNAs are not the only molecules that mediate gene expression in plants. These miRNAs may have been down regulated at this concentration because other pathways in the plant were responding to the aluminum oxide nanoparticle stress. The great amount of up regulation in expression of the nine miRNAs suggests that these miRNAs either play an alternative role in mediating stress response to aluminum oxide nanopartices in tobacco, or they mediate other responses as a result of aluminum oxide nanoparticle stress.

A study conducted by Reyes (2007) shows that miR159 plays a role in the response of plants to drought and during seed germination. During seed imbibitions, the levels of abscisic acid (ABA) decreases in order to allow the seeds to intake water and to begin germination [26]. Under abiotic stress conditions, such as the conditions in our experiment, the levels of ABA within the seeds could possibly stay elevated and as a result, the growth and development of seedlings would be stunted. ABA also is known to induce miR159 which targets MYB101 and MYB33 and suppresses their expression [26]. The consequence of MYB101 and MYB33 suppression is that it makes plants hyposensitive to ABA, therefore affecting their ability to germinate. miR159 is also up regulated in response to drought, therefore it is believed that miR159 up regulation helps seeds to sense the environment and prevents germination if the environmental conditions are not optimal. In our experiment, miR159 was up regulated approximately 5.8 fold in response to 1% Al2O3 nanoparticles and the seed germination rate for the same concentration of nanoparticles was lower than the other concentrations. It is a possibility that miR159 expression was up regulated in aluminum oxide exposed tobacco seedlings in order to mediate a similar pathway as described above, or that the up regulation of this miRNA plays an alternate role in allowing the mediation of tobacco seedlings to aluminum oxide nanoparticle stress.

The target site of miR162 is the Dicer-Like 1 protein that functions in the biogenesis of miRNAs in plants [5]. Dicer-Like 1 functions to cleave pri-miRNA sequences into pre-miRNA sequences that further undergo cleavage until they become mature miRNAs [3], [4]. In our experiment, the levels of miR162 were up regulated, which would suggest that the amount of miR162's target protein Dicer-Like 1 would decrease, consequently decreasing the amount of mature miRNAs that could be produced. In our study, we saw that the majority of the miRNAs that we screened for ended up being up regulated, not down regulated as suggested. This implies that miRNA162 may have an alternate role in mediating abiotic stress response to aluminum oxide nanoparticle stress.

miR167 functions mainly to target the transcription factors, Auxin Response Factor 6 and 8 (ARF), which helps to regulate the levels of the hormone auxin within a plant [5], [27]. Auxin is a plant hormone that plays a central role in growth and development through the regulation of reproductive organs and root development, therefore if plants were not as sensitive to auxin, it could explain the decreased root lengths that we observed in our experiment. Most likely miR167 plays an alternate role in mediating stress response to aluminum oxide nanoparticles.

A recent study has shown that over-expression of miRNA169 confers enhanced drought tolerance to tomatoes [20] through the reduction in activity of the stomata. By reducing the activity of stomata, the rate of transpiration was significantly reduced, therefore reducing the amount of water loss from the leaves. In our study, miR169 was up regulated, therefore it is possible that over expression of this miRNA may have a similar effect on water loss in tobacco. Whether up regulation of miR169 plays a role in stomata activity in a similar manner in tobacco, the up regulation of this miRNA suggests that it plays some role in mediating stress response to aluminum oxide nanoparticles. Further experiments would need to be performed to see if miR169 controls the movement of stomata in the same manner as it does in tomato.

Many miRNAs have been identified to mediate nutrient homeostasis in plants [28]. Our results show that miR395 was up regulated the most with a fold change of approximately 315 fold as compared to the control. miR395 is known to sense sulfate starvation and helps to assimilate sulfur in plants [29]. Sulfur is an important element in plants because it is found in amino acids, oligopeptides, vitamins, cofactors, and many secondary products [30], therefore the regulation of the amount of sulfur found within plants is important to monitor. miR395 helps to monitor this process because it targets several genes that are involved in sulfur assimilation [29]. When sulfur levels are low within plants, miR395 expression is greatly increased; therefore the results of our experiment indicate that the tobacco seedlings may have been undergoing sulfur starvation as a result of the up regulation in expression of miR395. If the seedlings were undergoing sulfur starvation, that would also explain why as the concentration of the aluminum oxide nanoparticles increased, the overall growth and development of the seedlings decreased. Because sulfur is a key element in so many biologically important molecules, the growth and development of the plants would be affected if the plants were starved for sulfur. It is also a possibility that miR395 plays a role in mediating another pathway in plants and helps to mediate stress response to aluminum oxide nanoparticle exposure through an alternative pathway.

miR396 has been shown to aid in both cell proliferation in *Arabidopsis* [31] and tolerance to drought in tobacco [19]. Our findings show that miR396 was up regulated 12 fold more than the basal expression levels in tobacco. In brief, the study conducted by Rodriguez et. al (2010) shows that *Arabidopsis* plants which have higher levels of miR396 also have higher levels of the transcription factor TCP4 and lower levels of GRFs (Growth Regulating Factors). Because of the increased miR396 levels leading to increased amounts of TCP4, cell proliferation is slowed and plant growth and development is affected as a result. Yang and Yu (2009) conducted a study to assess the effect of miR396 on tolerance to drought in tobacco [32]. They found that if miR396 was up regulated in tobacco, then the plants had a higher tolerance to drought through reducing the size of their leaves and by reducing their stomatal index. Based on these two studies, since miR396 was up regulated in the aluminum oxide nanoparticle exposed seedlings, it is possible that the growth and development of the seedlings was stunted due to a decrease in cell proliferation. It is also possible that the reason why the seedlings did not die is due to the fact that increased levels of miR396 helped in aiding the retention of water within the plants as shown in tobacco by Yang and Yu (2009). It is also a possibility that miR396 may have an alternate role in the response of tobacco seedlings to aluminum oxide nanoparticles.

miR397 was also significantly up regulated in our experiment. miR397 has been shown to play a role in drought stress response, nutrient deprivation, and copper homeostasis [33], [34] within plants. Copper serves as a critical cofactor for components of the electron transport chain, and as a cofactor for proteins involved in metabolism, the removal of reactive oxygen species, and in cell signaling. Since miR397 was up regulated, this suggests that the seedlings may possibly have been deprived of copper. If this was the case, copper starvation would explain the decrease in growth of the tobacco seedlings since copper plays a key role in many cellular processes. In this experiment, miR397 was up regulated 55 fold in the 1% Al_2_O_3_ concentration of nanoparticles; therefore we believe that this miRNA may play a role in the reponse of tobacco to aluminum oxide nanoparticles through a similar pathway as described above, or through an alternate pathway.

miR398 has also been shown to be up regulated in response to water deficit in *Medicago truncatula* [35]and has alsobeen shown to be down regulated in response to oxidative stress or low copper levels [23]. Another study has also shown that miR398 is up regulated in the presence of 1% sucrose [36]. Our seedlings were grown in media supplemented with 1% sucrose, therefore it is a possibility that miR398 could have been up regulated simply due to exposure to sucrose in our growth media. More likely, miR398 plays an alternate role in mediating stress to aluminum oxide nanoparticles in tobacco seedlings. More research would need to be conducted to elucidate the exact mechanism miR398 has in response to aluminum oxide nanoparticles exposure.

miR399 has been shown to play a key role in regulating phosphate starvation in plants [37]. Phosphate plays a key role in metabolism and signaling, therefore levels in plants needs to be highly regulated. In our experiment, miR399 was greatly up regulated (90 fold), third behind miR395, therefore the seedlings may possibly have been under the influence of phosphate starvation which would explain the decrease in growth and development of the seedlings. Because miR395 (sulfate regulation) and miR399 (phosphate regulation) were both up regulated the most in response to 1% Al2O3 nanoparticles, these two miRNAs may play a key role in response to stress to aluminum oxide nanoparticles exposure besides their known roles in sulfate and phosphate regulation.

Overall, we found through our study that Al_2_O_3_ nanoparticles have a negative impact on the growth and development of three week old tobacco seedlings. We saw an overall significant reduction in root length, average biomass, and leaf count of each tobacco seedling after being exposed to 0.1%, 0.5%, and 1% Al_2_O_3_ nanoparticles, but no significant change in the germination rates of the seedlings. We have not conducted any studies to show the exact mode of phytotoxicity of aluminum oxide nanoparticle exposure to tobacco seedlings. The two common possibilities are either the nanoparticles will adhere to the roots impeding any uptake of water and nutrients, or that the nanoparticles are uptaken and translocated within the plant therefore causing toxicity internally. Aluminum oxide exists commonly in the soil as aluminum is one of the most abundant elements found in the earth's crust. It is not known how much of the aluminum oxide found in the soil exists as nanoparticles, therefore more research needs to be conducted to determine if the increasing use of aluminum oxide nanoparticles in industry is drastically affecting the concentrations of aluminum oxide that is already present in the soil. More research also needs to be conducted in order to elucidate the mechanisms of aluminum oxide nanoparticle toxicity and to determine the exact mode of phytotoxicity in plants.

miRNA expression levels in tobacco were also altered as a result of exposure to aluminum oxide nanoparticles. In particular, miR395, miR398, and miR399 exhibited the greatest increase in fold changes of 315, 144, and 90, respectively. These miRNAs play a key role in nutrient starvation and resistance to drought, therefore this study suggests that these miRNAs may either play a similar role in nutrient starvation and resistance to drought as shown in other experiments or that these miRNAs play alternate unknown roles in response to stress caused by exposure to aluminum oxide nanoparticles. Our study analyzed the changes in expression levels of only twelve conserved miRNAs, therefore in order to identify novel tobacco miRNAs that may play a role in plant tolerance to aluminum oxide nanoparticle stress, other experiments such as high through-put sequencing will need to be performed. We found that aluminum oxide nanoparticles affected miRNA expression levels in tobacco and since they play a key role as gene regulators, miRNAs may play a vital role in tobacco tolerance to stress caused by aluminum oxide nanoparticle exposure. As a result, more research needs to be conducted to identify the roles of miRNAs in mediating plant stress responses in order to subsequently improve the ability of plants to withstand stresses in the environment.
